# ﻿The first two complete mitochondrial genomes for the genus *Anagyrus* (Hymenoptera, Encyrtidae) and their phylogenetic implications

**DOI:** 10.3897/zookeys.1206.121923

**Published:** 2024-07-05

**Authors:** Cheng-Hui Zhang, Hai-Yang Wang, Yan Wang, Zhi-Hao Chi, Yue-Shuo Liu, Guo-Hao Zu

**Affiliations:** 1 College of Horticulture and Landscape, Tianjin Agricultural University, Tianjin 300392, China Tianjin Agricultural University Tianjin China

**Keywords:** Encyrtid, genome structure, mitogenome, protein-coding genes, phylogenetic analyses, Tetracneminae

## Abstract

*Anagyrus*, a genus of Encyrtidae (Hymenoptera, Chalcidoidea), represents a successful group of parasitoid insects that attack various mealybug pests of agricultural and forestry plants. Until now, only 20 complete mitochondrial genomes have been sequenced, including those in this study. To enrich the diversity of mitochondrial genomes in Encyrtidae and to gain insights into their phylogenetic relationships, the mitochondrial genomes of two species of *Anagyrus* were sequenced, and the mitochondrial genomes of these species were compared and analyzed. Encyrtid mitochondrial genomes exhibit similarities in nucleotide composition, gene organization, and control region patterns. Comparative analysis of protein-coding genes revealed varying molecular evolutionary rates among different genes, with six genes (*ATP8*, *ND2*, *ND4L*, *ND6*, *ND4* and *ND5*) showing higher rates than others. A phylogenetic analysis based on mitochondrial genome sequences supports the monophyly of Encyrtidae; however, the two subfamilies, Encyrtinae and Tetracneminae, are non-monophyletic. This study provides valuable insights into the phylogenetic relationships within the Encyrtidae and underscores the utility of mitochondrial genomes in the systematics of this family.

## ﻿Introduction

Encyrtidae is a large hymenopteran family in the superfamily Chalcidoidea, comprising 518 known genera, of which 495 are recognized as valid (totaling more than 4830 species), along with 23 fossil genera (26 species) worldwide (Simutnik et al. 2022; Simutnik et al. 2023; Simutnik and Perkovsky 2023; Wang et al. 2023). The genus *Anagyrus* Howard, 1986 is one of the largest genera in Encyrtidae, comprising 289 valid species (Noyes 2019). This genus was established by Howard and Ashmead (1896) based on the type species, *Anagyrusgreeni* Howard, 1896. Diagnostics for the genus include a broadened, flattened scape (normally 2–3× as long as broad), funicle segments longer than broad, occipital margin normally quite sharp but often rounded, postmarginal vein normally not longer than the stigma vein, and ovipositor at least half the length of the mid tibia (Noyes 1980; Noyes and Hayat 1994). *Anagyrus* species are primary parasitoids of Pseudococcidae; for example, *Anagyrusgalinae* has been utilized in classical biocontrol and integrated pest management of *Trionymuscopiosus* (Japoshvili and Hansen 2015; Noyes 2019).

Insect mitochondrial genomes are usually small, circular molecules containing 37 genes: 13 protein-coding genes (PCGs), two ribosomal RNA genes (rRNAs), and 22 transfer RNA genes (tRNAs), as well as a large non-coding element known as the A+T-rich or control region (CR), which regulates transcription and replication (Wolstenholme 1992a, 1992b; Boore 1999; Cameron 2014). Due to their distinct characteristics, including gene-content conservation, maternal inheritance, and rapid evolutionary rate, mitogenome sequences serve as valuable molecular markers for various evolutionary studies (Boore 1999; Krzywinski et al. 2006). Although the mitochondrial genome of Chalcidoidea exhibits structural resemblance to other insects, significant rearrangements characterize it, along with a relatively high A+T content in its sequence composition, deviating from the presumed ancestral pattern (Brown et al. 1979; Cameron and Whiting 2008).

The exploration of hymenopteran mitochondrial genomes commenced with the sequencing of *CYTB* and *ATP8* genes of *Apismellifera*, and it was not until 1993 that the first complete mitochondrial genome was deciphered (Crozier and Crozier 1992, 1993). The first comprehensive phylogenetic analysis of Chalcidoidea based on molecular data was conducted using 18S and 28S rDNA (Munro et al. 2011). Subsequently, Heraty et al. (2013) conducted an in-depth exploration of the phylogenetic relationships within Chalcidoidea based on both morphological and molecular data. Zhang et al. (2020a) further reconstructed the phylogenetic relationships within Chalcidoidea using transcriptome data, providing valuable insights for achieving more accurate phylogenetic relationships. Recently, Cruaud et al. (2024) conducted a comprehensive phylogenetic study using data from PCGs and ultra-conserved elements (UCEs), while Zhu et al. (2023) conducted a comprehensive phylogenetic study using 139 mitochondrial genomes from the main clades of Chalcidoidea. These studies have significantly advanced our understanding of the phylogenetic relationships within Chalcidoidea. However, to obtain a more accurate reconstruction of evolutionary relationships, it is necessary to expand the sampling range to include more understudied species. This approach will help construct a more comprehensive and precise phylogenetic tree, revealing deeper levels of phylogenetic relationships. Additionally, integrating different types of data, such as rDNA genes, mitochondrial genomes, and UCEs, is crucial. By comprehensively utilizing morphological, biological, and molecular data and conducting multidimensional phylogenetic analyses, we can improve the accuracy of classification and phylogenetic research. Such integrative approaches will provide a more robust framework for understanding the evolutionary relationships within Hymenoptera.

At present, there are only morphology-based classification systems for Encyrtidae (Noyes and Hayat 1984, 1994; Trjapitzin 1989), lacking auxiliary verification from molecular data, particularly from the mitochondrial genome (mitogenome). Consequently, the monophyly and phylogenetic relationships of Encyrtidae have been controversial for a long time. Problems that are difficult to distinguish in taxonomy indicate the requirement for using various molecular data to understand the systematic position and the monophyly of Encyrtidae in Chalcidoidea. Mitogenome data seem sufficient to solve these problems (Wei et al. 2010; Li et al. 2016; Liu et al. 2023). There are currently only 1291 complete mitochondrial genomes of Hymenoptera on GenBank, and the number of encyrtid genomes is small (Sayers et al. 2024). This limited data negatively impacts our ability to resolve potential systematic ambiguity within Encyrtidae.

In this study, we conducted the sequencing and annotation of the mitogenomes of *Anagyrusgalinae* (accession number: OR652687) and *Anagyrusjenniferae* (accession number: OR790122), analyzing their respective characteristics. In addition, we reconstructed the molecular phylogenetic relationships of these two new mitochondrial genomes and other species of Encyrtidae. The molecular data presented in this study will contribute to a better understanding of the characteristics of the Encyrtidae mitogenome. Further, a phylogenetic analysis was performed, including 19 uploaded mitogenomes together with our newly acquired data, which represented Encyrtidae. The goal of our study was to place two new species of *Anagyrus* within the context of the known mitogenome diversity of Encyrtidae by performing mitogenomic and phylogenetic analyses.

## ﻿Materials and methods

### ﻿Sample collection, DNA extraction and sequencing

The specimens, *A.galinae* and *A.jenniferae*, were collected from Tianjin Agricultural University (39°5′21″N, 117°5′38″E), Xiqing District, Tianjin City, China, in September 2022. Freshly collected specimens were promptly immersed in 100% ethanol for initial preservation and subsequently stored at −40 °C in the Insect Herbarium of Tianjin Agricultural University. Following morphological identification, total DNA from each specimen was extracted from the body, excluding the abdomen, using the DNeasy Blood & Tissue Kit (Qiagen, Hilden, Germany) according to the manufacturer’s instructions. The purity and concentration of the extracted total DNA were assessed through 1% agarose gel electrophoresis and optical density value detection. The total DNA of two encyrtids underwent sequencing using the Illumina NovaSeq 6000 platform with a 350 bp insert size and a paired-end 150 bp sequencing strategy. Sequencing was conducted by Novogene Co., Ltd. (Beijing, China).

### ﻿Mitogenome assembly, annotation and analysis

After initial data acquisition, with adapter sequences removed, additional filtering was carried out using fastp 0.23.4 (Chen et al. 2018) to filter low-quality reads (quality value <30), ensuring that each sample retained clean data of no less than 4 Gb. The software MitoZ v. 3.6 (Meng et al. 2019) and GetOrganelle v. 1.7.7.0 (Jin et al. 2020) were used for the de novo assembly of mitogenomes. Homologous sequences of other Encyrtidae species from GenBank were used for comparison, and the mitogenomes were annotated using the Mitos WebServer (Donath et al. 2019). The secondary structures of tRNAs were predicted using Mitos WebServer and further visualized using VARNA v. 3.9 (Darty et al. 2009). The structures of the mitochondrial genome were mapped using the online tool CGview Server. The nucleotide composition and relative synonymous codon usage (RSCU) of protein-coding genes were calculated and analyzed by MEGA v. 11.0.13 (Tamura et al. 2021). The skew analysis of nucleotide composition was calculated using the formulas: AT-skew = (A−T)/(A+T) and GC-skew = (G−C)/(G+C), where A, T, G and C were the base contents of the same chain (Perna and Kocher 1995; Hassanin et al. 2005). The nonsynonymous mutation rate (Ka) and synonymous mutation rate (Ks) of protein coding genes were calculated using DnaSP 6.12.03 (Rozas et al. 2017). Tandem repeats in the CR were identified by Tandem Repeats Finder (Benson 1999).

### ﻿Molecular phylogenetic analyses

A total of 21 mitogenomes from two families of Chalcidoidea, including 20 Encyrtidae species and a Aphelinidae species as outgroup, were used for the phylogenetic analysis (Table 1). The phylogenetic trees were reconstructed using both maximum-likelihood (ML) and Bayesian-inference (BI) methods. For this, each PCG was individually aligned using the MAFFT 7 online service with the L-INS-i strategy, followed by optimization using MACSE (Ranwez et al. 2018; Katoh et al. 2019). The individual PCG alignments were trimmed using GBlocks and concatenated into a PCG dataset using PhyloSuite v. 1.2.3 (Talavera and Castresana 2007; Zhang et al. 2020b). The best nucleotide substitution model was obtained using ModelFinder v. 2.2.0 with Bayesian Information Criterion (BIC) (Kalyaanamoorthy et al. 2017). BI analysis was performed using MrBayes v. 3.2.7a with four chains (Ronquist et al. 2012). Two independent runs of 2,000,000 generations were carried out with sampling every 1,000 generations. The first 25% of trees were discarded as burn-in. After the average standard deviation of split frequencies fell below 0.01 and the potential scale reduction factor (PSRF) approached 1.0, stationarity was assumed. ML analysis was performed using IQ-TREE v. 2.2.0 (Nguyen et al. 2015) under the standard bootstrap approximation approach with 1,000 replicates.

**Table 1.** GenBank accession numbers of species used in phylogenetic reconstruction and their original publications.

Superfamily	Family	Species	Accession Number	References
Chalcidoidea	Aphelinidae	*Encarsiaformosa*	MG813797	Zhu et al. 2018
	Encyrtidae	*Aenasiusarizonensis*	NC_045852	Ma et al. 2019
		*Anagyrusgalinae*	OR652687	This study
		*Anagyrusjenniferae*	OR790122	This study
		*Blastothrixspeciosa*	NC_082111	Unpublished
		*Cheiloneuruschinensis*	NC_084192	Unpublished
		*Cheiloneuruselegans*	NC_071192	Unpublished
		*Diaphorencyrtusaligarhensis*	NC_046058	Du et al. 2019
		*Encyrtusaurantii*	OR120384	Unpublished
		*Encyrtuseulecaniumiae*	NC_051459	Rudoy et al. 2022
		*Encyrtusinfelix*	NC_041176	Xiong et al. 2019
		*Encyrtusrhodococcusiae*	NC_051460	Rudoy et al. 2022
		*Encyrtussasakii*	NC_051458	Rudoy et al. 2022
		*Exoristobiaphilippinensis*	NC_084171	Unpublished
		*Lamennaisiaambigua*	NC_082113	Unpublished
		*Lamennaisianobilis*	NC_061411	Unpublished
		*Leptomastideabifasciata*	OR790123	Unpublished
		*Metaphycuseriococci*	NC_056349	Zhou et al. 2021
		*Ooencyrtusplautus*	NC_068223	Xing et al. 2022
		*Psyllaephagus* sp.	OP787025	Unpublished
		*Tassoniagloria*e	NC_082112	Unpublished

## ﻿Results

### ﻿Mitogenome organization and nucleotide composition

**Figure 1.**
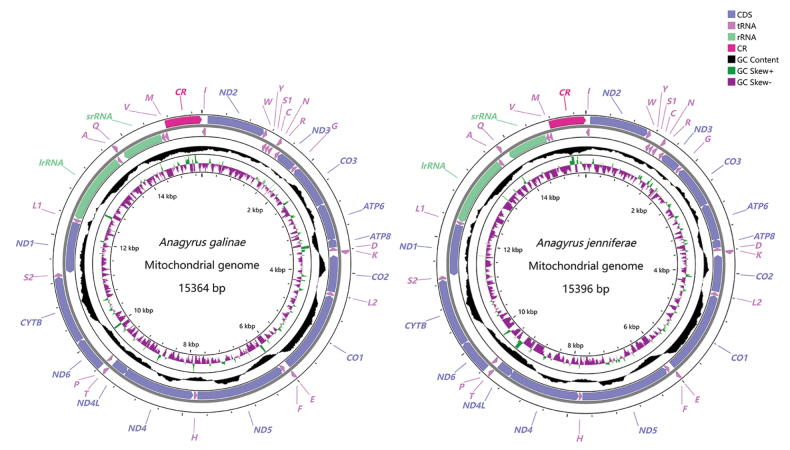
Circular map of the mitochondrial genome of *Anagyrusgalinae* and *Anagyrusjenniferae*.

The assembled mitochondrial genome of *A.galinae* was a 15,364 bp, and the *A.jenniferae* mitochondrial genome was 15,396 bp, which both had the same gene organization, including 13 PCGs, 22 tRNAs, two rRNAs and a control region located between *trnM* and *trnI* (Fig. 1). For the mitogenomes of two species, the majority strand (J-strand) encodes 10 PCGs (*ND3*, *CO3*, *ATP6*, *ATP8*, *CO2*, *CO1*, *ND5*, *ND4*, *ND4L*, *ND1*), 15 tRNAs (*trnI*, *trnY*, *trnS1*, *trnC*, *trnR*, *trnG*, *trnD*, *trnL2*, *trnF*, *trnH*, *trnP*, *trnL1*, *trnA*, *trnV*, *trnM*) and 2 rRNAs (*lrRNA*, *srRNA*), while the remaining three PCGs (*ND2*, *ND6*, *CYTB*) and seven tRNAs (*trnW*, *trnN*, *trnK*, *trnE*, *trnT*, *trnS2*, *trnQ*) are located on the minority strand (Table 2). Two mitogenomes both obtained 13 overlapping nucleotides, and up to 53 bp ranging from 1 to 16 bp. The longest overlap was located between *CO1* and *trnE* in *A.jenniferae*. There were 17 and 16 intergenic spacers each from *A.galinae* and *A.jenniferae*, totaling 171 bp and 115 bp, ranging 1 to 77 bp and 1 to 27 bp, respectively.

**Table 2.** Gene organization of the mitochondrial genomes of *Anagyrusgalinae* and *Anagyrusjenniferae*.

Gene	Direction		Anticodon	*Anagyrusgalinae*					*Anagyrusjenniferae*				
			Position	Length	Start codon	Stop codon	Intergenic Nucleotides	Position		Length	Start codon	Stop codon	Intergenic Nucleotides
*trnI*	−	GAU	1–70	70				1–67		67			
*ND2*	+		98–1087	990	ATT	TAA	27	74–1081		1008	ATT	TAA	6
*trnW*	+	UCA	1087–1149	63			−1	1080–1146		67			-2
*trnY*	−	GUA	1155–1221	67			5	1148–1212		65			1
*trnS1*	−	UCU	1222–1280	59			0	1216–1275		60			3
*trnC*	−	GCA	1283–1348	66			2	1293–1361		69			17
*trnN*	+	GUU	1369–1434	66			20	1368–1431		64			6
*trnR*	−	UCG	1433–1497	65			−2	1439–1504		66			7
*ND3*	−		1498–1842	345	ATT	TAA	0	1505–1858		354	ATA	TAA	0
*trnG*	−	UCC	1843–1906	64			0	1856–1919		64			-3
*CO3*	−		1911–2714	804	ATG	TAA	4	1925–2710		786	ATG	TAA	5
*ATP6*	−		2715–3387	673	ATG	T	0	2710–3383		674	ATG	TA	-1
*ATP8*	−		3381–3542	162	ATT	TAA	−7	3377–3538		162	ATC	TAA	-7
*trnD*	−	GUC	3543–3608	66			0	3539–3602		64			0
*trnK*	+	UUU	3612–3683	72			3	3606–3676		71			3
*CO2*	−		3688–4365	678	ATT	TAG	4	3678–4355		678	ATT	TAA	1
*trnL2*	−	UAA	4369–4434	66			3	4365–4428		64			9
*CO1*	−		4440–5987	1548	ATT	TAA	5	4431–5969		1539	ATG	TAA	2
*trnE*	+	UUC	5972–6036	65			−16	5972–6034		63			2
*trnF*	−	GAA	6036–6102	67			−1	6034–6099		66			-1
*ND5*	−		6102–7769	1668	ATA	TAA	−1	6099–7763		1665	ATT	TAG	-1
*trnH*	−	GUG	7767–7833	67			−3	7764–7829		66			0
*ND4*	−		7844–9169	1326	ATG	TAG	10	7829–9156		1328	ATG	TA	-1
*ND4L*	−		9163–9450	288	ATT	TAA	−7	9150–9437		288	ATT	TAA	-7
*trnT*	+	UGU	9453–9518	66			2	9440–9505		66			2
*trnP*	−	UGG	9520–9582	63			1	9506–9574		69			0
*ND6*	+		9584–10151	568	ATG	T	1	9575–10143		569	ATG	TA	0
*CYTB*	+		10152–11300	1149	ATG	TAA	0	10143–11285		1143	ATG	TAA	-1
*trnS2*	+	UGA	11300–11365	66			−1	11290–11354		65			4
*ND1*	−		11356–12291	936	ATT	TAG	−10	11345–12283		939	ATA	TAG	-10
*trnL1*	−	UAG	12292–12358	67			0	12284–12348		65			0
*lrRNA*	−		12364–13674	1311			5	12353–13654		1302			4
*trnA*	−	UGC	13682–13744	63			7	13651–13719		69			-4
*trnQ*	+	UUG	13761–13831	71			16	13797–13864		68			77
*srRNA*	−		13831–14602	772			−1	13891–14646		756			26
*trnV*	−	UAC	14602–14669	68			−1	14646–14710		65			-1
*trnM*	−	CAU	14668–14735	68			−2	14709–14770		62			-2
CR			14736–15364	629			0	14771–15396		626			0

The nucleotide composition of the mitogenome from *A.galinae* was biased toward A and T, with 83.12% of A+T content (A = 45.12%, T = 38.00%, C = 10.82%, G = 6.05%), A+T content was 82.94%, 87.20% in PCGs and rRNAs, respectively. The nucleotide composition of the mitogenome from *A.jenniferae* was biased toward A and T, with 82.64% of A+T content (A = 46.41%, T = 36.23%, C = 11.33%, G = 6.02%), A+T content was 82.32%, 85.20% in PCGs and rRNAs, respectively. The values of AT-skew and GC-skew were often used to indicate the nucleotide composition of the mitochondrial genome. In this study, the nucleotide features of two new mitogenomes were investigated by calculating the percentages of AT-skew and GC-skew (Table 3). The skew analysis showing the mitogenome of *A.galinae* had a positive AT-skew (0.086) and a negative GC-skew (−0.283), and the mitogenome of *A.jenniferae* had a positive AT-skew (0.123) and a negative GC-skew (−0.306).

**Table 3.** Nucleotide features of the mitochondrial genome across *Anagyrusgalinae* and *Anagyrusjenniferae*.

Feature	Length (bp)	T%	C%	A%	G%	A+T%	AT-Skew	GC-Skew
Whole genome	15364/15396	38.00/36.23	10.82/11.33	45.12/46.41	6.05/6.02	83.12/82.64	0.086/0.123	−0.283/−0.306
*ATP6*	673/674	46.66/47.63	7.43/8.01	34.92/34.27	11.00/10.09	81.58/81.90	−0.144/−0.163	0.194/0.115
*ATP8*	162/162	48.77/48.77	4.32/4.94	43.83/36.42	3.09/9.88	92.59/85.19	−0.053/−0.145	−0.167/0.333
*CO1*	1524/1539	45.41/46.39	10.37/10.98	29.86/27.23	14.37/15.40	75.26/73.62	−0.207/−0.260	0.162/0.167
*CO2*	678/678	45.58/45.72	8.55/8.41	33.04/33.19	12.83/12.68	78.61/78.91	−0.159/−0.159	0.200/0.203
*CO3*	804/786	46.64/49.75	7.84/8.52	32.21/29.90	13.31/11.83	78.86/79.64	−0.183/−0.249	0.259/0.163
*CYTB*	1149/1143	43.69/41.91	14.36/14.7	32.64/34.82	9.31/8.57	76.33/76.73	−0.145/−0.092	−0.213/−0.263
*ND1*	936/939	46.47/48.35	7.05/6.71	32.37/31.31	14.1/13.63	78.85/79.66	−0.179/−0.214	0.333/0.340
*ND2*	990/1008	50.10/47.52	9.19/9.62	37.58/39.19	14.10/13.63	87.68/86.71	−0.143/−0.096	−0.492/−0.448
*ND3*	345/351	51.01/52.99	5.22/5.41	33.91/31.34	9.86/10.26	84.93/84.33	−0.201/−0.257	0.308/0.309
*ND4*	1326/1328	50.08/52.41	4.98/5.20	34.01/30.20	10.94/12.20	84.09/82.61	−0.191/−0.269	0.374/0.403
*ND4L*	288/288	53.13/53.82	2.78/2.08	34.03/36.46	10.07/7.64	87.15/90.28	−0.219/−0.192	0.568/0.571
*ND5*	1665/1665	50.81/51.11	5.77/5.77	33.09/32.61	10.33/10.51	83.90/83.72	−0.211/−0.221	0.284/0.292
*ND6*	568/569	46.13/45.34	8.45/10.54	42.25/41.48	3.17/2.64	88.38/86.82	−0.044/−0.045	−0.455/−0.600
*srRNA*	772/756	44.30/44.84	4.15/4.10	43.52/40.08	8.03/10.98	87.82/84.92	−0.009/−0.056	0.319/0.456
*lrRNA*	1311/1302	44.55/46.08	4.27/4.15	42.03/39.40	9.15/10.37	86.58/85.48	−0.029/−0.078	0.364/0.429
CR	629/626	42.61/40.57	7.15/8.47	46.26/48.72	3.98/2.24	88.87/89.29	0.041/0.091	−0.285/−0.582

### ﻿Protein-coding genes and codon usage

**Figure 2.**
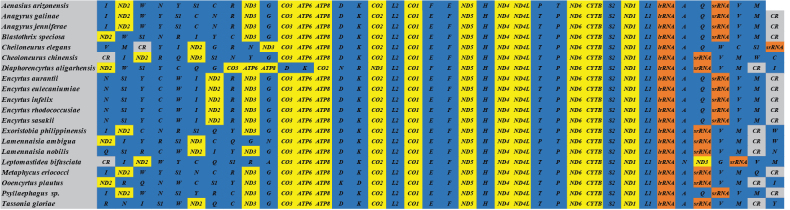
Gene order of mitochondrial genomes of different Encyrtidae species.

By comparing the known mitochondrial genome structure of Encyrtidae, we found that the sequence of 13 PCGs was consistent, except for *ND3* rearranged in *Diaphorencyrtusaligarhensis* and *Leptomastideabifasciata*. The sequence of PCGs in these mitochondrial genomes were the same (Fig. 2). Additionally, this arrangement is consistent with the mitochondrial gene order in other Encyrtidae, which is also consistent with inferred ancestry.

The total lengths of 13 PCGs are 11,108 bp in *A.galinae*, 11,130 bp in *A.jenniferae*. In these mitochondrial genomes, the length of each PCG ranges from 162 bp (*ATP8*) to 1665 bp (*ND5*). Two mitogenomes of *Anagyrus* exhibited similar start and stop codons. All the initiation codons of PCGs were ATN (ATA, ATG and ATT). Three kinds of stop codons existed on the new mitogenomic sequences: TAA, TAG and truncated termination codons (TA existed on *ATP6*, *ND4*, *ND6* in *A.jenniferae*, T existed on *ATP6*, *ND6* in *A.galinae*), TAA were the most frequently used. Truncated termination codons are commonly used in metazoan mitogenomes, which could be completed by post-transcriptional poly-adenylation (Ojala et al. 1981).

**Figure 3.**
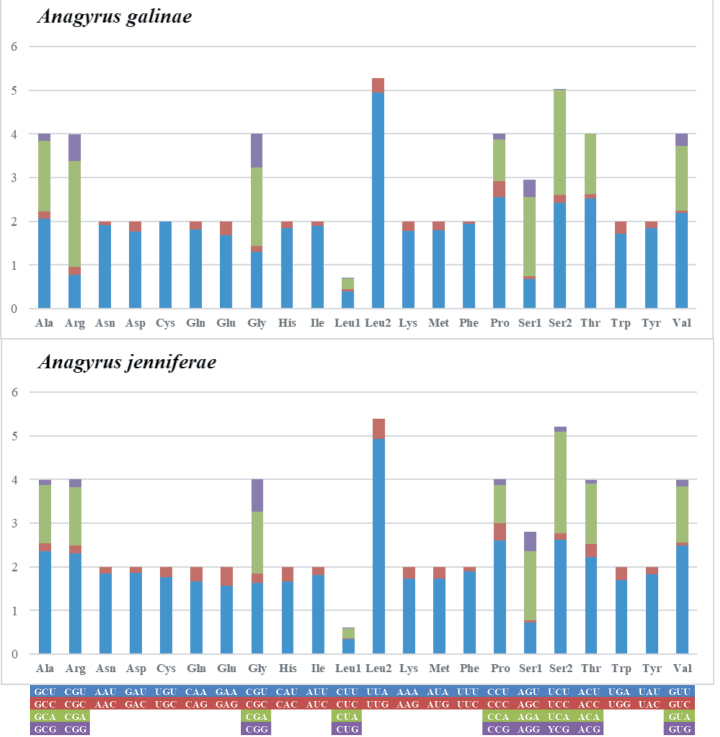
Relative synonymous codon usage in mitochondrial genomes of *Anagyrusgalinae* and *Anagyrusjenniferae*.

The codon UUA (Leu2) was the most commonly used in both mitogenomes. Mitochondrial protein coding genes have obvious bias towards A and T, and for mitochondrial protein-coding gene of *A.galinae* the three most frequently used codons were UUA (Leu2) 469 times, AUU (Ile) 440 times and UUU (Phe) 432 times. For *A.jenniferae*, the three most used codons were UUA (Leu2) 463 times, UUU (Phe) 431 times and AUU (Ile) 393 times. Mitochondrial protein-coding genes in Encyrtidae prefer A and U in the third codon, which is like some hymenopteran insects (Fan et al. 2017; Peng et al. 2017). The RSCU values of *A.galinae* and *A.jenniferae* are shown in Fig. 3.

**Figure 4.**
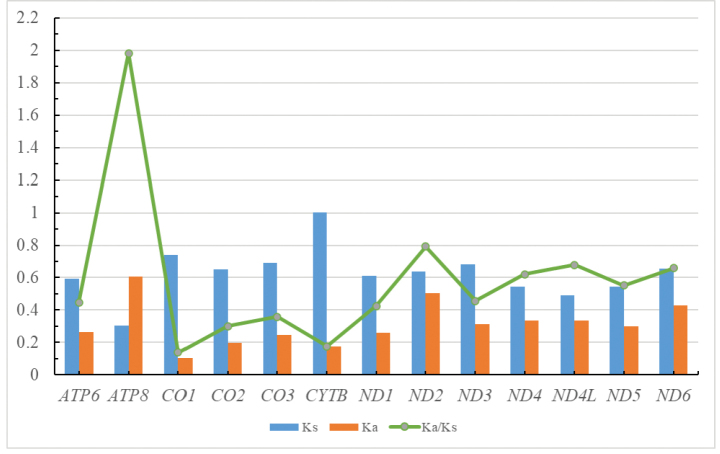
Evolutionary rates of protein-coding genes in the mitochondrial genomes of Encyrtidae.

In this study, based on 20 mitochondrial genomes of Encyrtidae, DnaSP was used to calculate the non-synonymous substitution rate, synonymous substitution, and Ka/Ks ratio of 13 PCGs in the mitochondrial genome and then to compare the evolution rate between genes (Fig. 4). The results showed that among the 13 protein-coding genes in the mitochondrial genome of encyrtids, *CYTB* had the highest Ks, whereas *ATP8* had the highest Ka and Ka/Ks value, and *ATP8* had the largest variation and *COI* had the slowest evolution rate. The evolution rate of 13 genes was in the order of *ATP8* > *ND2* > *ND4L* > *ND6* > *ND4* > *ND5* > *ND3* > *ATP6* > *ND1* > *CO3* > *CO2* > *CYTB* > *CO1*.

Ka/Ks values of 12 PCGs (all PCGs except *ATP8*) were far lower than 1.0, indicating that they were subject to purifying selection, a phenomenon first discovered in Chalcidoidea. In addition, the Ka/Ks value of *ATP8* is higher than 1.0, higher value of *ATP8* was also found in other species (Ma et al. 2019; Jia et al. 2020; Xu et al. 2021). The reason for this phenomenon may be that the evolution speed of a gene is related to its function (Wang et al. 2011).

### ﻿Transfer RNA genes, ribosomal RNA genes, and control region

The mitochondrial genomes of the two species both included 22 tRNA genes, and the total lengths of the tRNAs of *A.galinae* and *A.jenniferae* are 1455 bp and 1445 bp, respectively. The length of tRNA genes in two *Anagyrus* species ranged from 59 to 72 bp. The secondary structures of the 22 tRNAs of the two species are shown in Suppl. materials 1, 2. The 22 tRNA genes in the mitochondrial genome are identical with the anticodon of tRNA corresponding to the mitochondrial genome of other Hymenoptera, except that *trnL* and *trnS* have two tRNA structures, and the others only have one corresponding tRNA structure. Most tRNAs could be folded into a typical clover-leaf structure, except for *trnS1* which lost a dihydrouridine (DHU) arm and became a simple loop. A lack of the DHU arm in *trnS1* was found in the mitochondrial genomes of most insects (Dowton et al. 2002). Changes in the length of the DHU and TΨC arms led to differences in the size of the tRNA sequence (Shao et al. 2001). In addition, the anticodon of *trnS1* became UCU instead of the more common GCU. In addition to typical Waston-Crick pairings (A-U and G-C), G-U pairings also exist, which are called atypical pairings or wobble base pairs. A total of 30 mismatched base pairs were found in the arm structures of the tRNAs.

Hymenopteran mitochondria have a high rearrangement rate, which mainly occurs in A+T-rich regions, *ND2*, *ND2*-*CO2*, *CO2*-*ATP8*, and *ND3*-*ND5* regions (Wei 2009). The gene arrangement of the suborder Symphyta was conserved and less rearranged than that of suborder Apocrita. However, there are a large number of rearrangements in the Apocrita, including displacement, inversion in situ, and ectopic inversion (Song 2015; Zhao et al. 2021). The rearrangement of mitochondrial genomes in Encyrtidae species was compared (Fig. 2), and the rearrangement was mainly found in tRNA genes. The rearrangement of tRNA occurred at many sites, and the pattern was complicated. Except that *trnD*-*trnK* (*trnK*-*trnD* in *Ooencyrtusplautus*), *trnL2*, *trnE*-*trnF* (*trnF*-*trnE* in *Aenasiusarizonensis* and *Diaphorencyrtusaligarhensis*), *trnH*, *trnT*-*trnP* (*trnP*-*trnT* in *Aenasiusarizonensis* and *Lamennaisiaambigua*), *trnS2* and *trnL1* are stable between *ATP8* to *lrRNA*, there was no exclusion, and the other tRNA genes had been rearranged.

As for the rRNAs of two *Anagyrus* species, both *lrRNA* and *srRNA* genes are encoded on the N-strand and have a heavy AT nucleotide bias. The lengths of *lrRNA* and *srRNA* in *A.galinae* are 1311 bp and 772 bp, with the different A+T contents of 86.58% and 87.82%, and in *A.jenniferae* are 1302 bp and 756 bp, with the different A+T contents of 85.48% and 84.92%.

**Figure 5.**
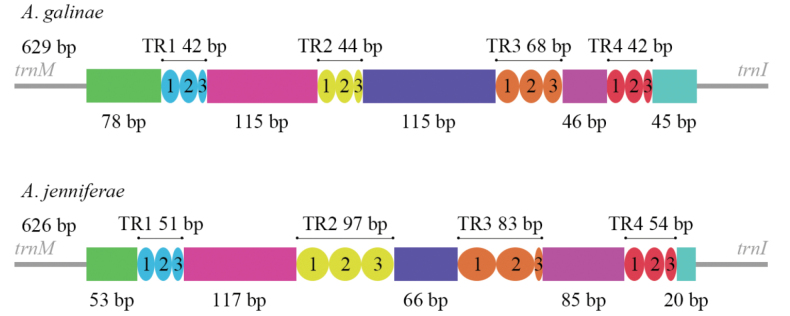
Control region structure of two *Anagyrus* species. TR, tandem repeat.

In the mitogenome, the largest non-coding region is normally the A+T-rich region, also known as the control region, which regulates the replication and transcription of mitochondrial DNA (Boore 1999; Cameron 2014). In the mitogenomes of the two *Anagyrus* species sequenced in this study, the CR is located between *trnM* and *trnI* (Fig. 5). The length of the CR is 629 bp in *A.galinae* and 626 bp in *A.jenniferae*. The A+T content is 88.87% and 89.29% in the CR of *A.galinae* and *A.jenniferae*. Analysis of AT-skew and CG-skew indicates that both *Anagyrus* species exhibit A and C usage bias. Three structural elements were found in each CR of two *Anagyrus* species: (1) a leading sequence adjacent to *trnM*; (2) four tendem repeats (TPs); (3) the remaining area of the control region.

### ﻿Phylogenetic relationships

**Figure 6.**
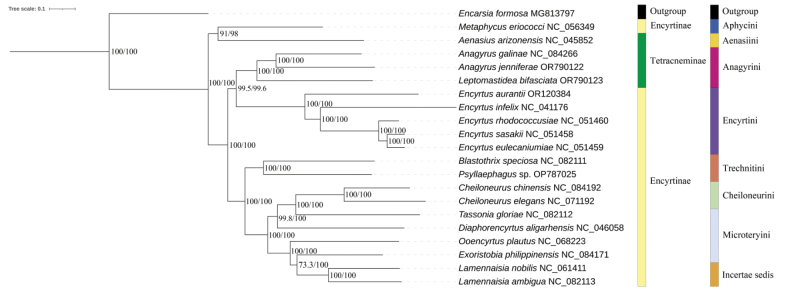
Phylogenetic tree of Encyrtidae based on nucleotide sequence of PCGs. Numbers at the nodes are Bayesian posterior probabilities (left) and ML bootstrap values (right). Each color block represents the corresponding family and tribe.

The phylogenetic analysis of the concatenated dataset was conducted using BI and ML, which were shown in Fig. 6. With *Encarsiaformosa* as an outgroup, the phylogenetic trees of Encyrtidae were constructed based on 13 protein-coding gene sequences of the 21 mitochondrial genomes, including NCBI data and the two newly sequenced *Anagyrus* genomes reported in this study.

The result of maximum-likelihood and Bayesian analysis both indicate that the taxonomic relationship of each genus of Encyrtidae is (*Metaphycus* + *Aenasius*) + (((*Anagyrus* + *Leptomastidea*) + *Encyrtus*) + ((*Blastothrix* + *Psyllaephagus*) + (((*Cheiloneurus* + *Tassonia*) + *Diaphorencyrtus*) + (*Ooencyrtus* + (*Exoristobia* + *Lamennaisia*))))).

Overall, the phylogenetic trees reconstructed by both methods indicate that species belonging to the same tribe are clustered into one or adjacent clades, while species belonging to the same genus are clustered into the same clade, consistent with the morphological classification system. At the subfamily level, according to the morphological classification system, Encyrtidae is divided into two subfamilies: Tetracneminae and Encyrtinae. *Aenasius*, *Anagyrus*, and *Leptomastidea* all belong to Tetracneminae, while the remaining genera belong to Encyrtinae. However, in the phylogenetic trees reconstructed in this study, the results of both methods show that, except for *Encyrtus* and *Metaphycus*, Encyrtidae is divided into two main parts, which essentially conforms to the morphological classification system. *Metaphycus* and *Aenasius* form a monophyletic clade as sister groups, which is consistent with the previous phylogenetic results (Zhao et al. 2021; Xing et al. 2022).

While the *Anagyrus* species were not clustered on one branch with *Aenasiusarizonensis* but clustered with *Encyrtus*, this may be due to different dietary habits. The five genera *Metaphycus*, *Aenasius*, *Anagyrus*, *Leptomastidea*, and *Encyrtus* exclusively parasitize scale insects within Hemiptera. In contrast, other species of Encyrtinae have a broader host range, including species from Lepidoptera, Diptera, Coleoptera, Hymenoptera, and more families within Hemiptera (Noyes 2019). Specifically, *Anagyrusjenniferae* parasitizes *Phenacoccusindicus*, *Anagyrusgalinae* parasitizes *Trionymuscopiosus*, and *Leptomastideabifasciata* parasitizes *Phenacoccusaceris* and *Planococcusvovae* (Noyes and Hayat 1994; Japoshvili and Hansen 2015; Trjapitzin 1989; Zhang and Xu 2009). These Anagyrini species, which exclusively parasitize the Pseudococcidae, form a distinct clade in both phylogenetic trees. The hosts of *Encyrtussasakii* include *Takahashiajaponica* and *Eulecaniumkuwanai*; *Encyrtuseulecaniumiae* parasitizes *Eulecaniumkuwanai* and *Eulecaniumgiganteum*; *Encyrtusrhodococcusiae* targets *Rhodococcussariuoni*; and *Encyrtusinfelix* parasitizes *Ceroplastesdestructor*, *Saissetiacoffeae*, and *Saissetiaoleae* (Trjapitzin 1989; Öncüer 1991; Noyes and Hayat 1994; Zhang and Huang 2001; Gupta and Poorani 2009; Wang et al. 2016), which were exclusively parasitize the Coccidae. Additionally, *Encyrtusaurantii* can parasitize members of the Coccidae (*Saissetiacoffeae*), Eriococcidae (*Eriococcusbuxi*), and Pseudococcidae (*Planococcuscitri*) (Hayat et al. 2003). Consequently, in the phylogenetic trees, the clustering of Anagyrini and Encyrtini species together in the phylogenetic analysis might be attributed to the close genetic relationship between Coccidae and Pseudococcidae (Cook et al. 2002). This phenomenon also indicates the need for further mitochondrial genome sequencing of Encyrtidae species to obtain a more accurate classification status.

## ﻿Discussion

In this study, we determined two newly sequenced mitogenomes, which are from *A.galinae* and *A.jenniferae*, then found them consistent with previously reported mitogenomes of Encyrtidae. Two new mitogenomes exhibited quite similar features in the genome size, base content, AT nucleotide bias, AT-skew, GC-skew, codon usage of protein genes, secondary structure of tRNAs and gene rearrangement. The BI and ML phylogenetic analysis among the major lineages based on the concatenated datasets yielded well-resolved topologies with moderate to high support for most branches. These results provide a relatively holistic framework and valuable data toward the future resolution of phylogenetic relationships in Encyrtidae. This study provided insights into the phylogenetic relationships of certain taxa within Encyrtidae, the limited sample size and scarcity of molecular evidence remain challenges. Therefore, future studies should aim to augment the number of sampled species and expand the dataset of mitochondrial genomes, utilizing a broader range of data for robust phylogenetic analysis and a comprehensive assessment of the taxonomic status within Encyrtidae.

## ﻿Additional information

### Author ORCIDs

Cheng-Hui Zhang https://orcid.org/0000-0001-8234-0903

Hai-Yang Wang https://orcid.org/0009-0007-5665-2111

Yan Wang https://orcid.org/0000-0002-5001-975X

Zhi-Hao Chi https://orcid.org/0009-0006-0447-6948

Guo-Hao Zu https://orcid.org/0000-0002-9892-2171

### Data availability

Data presented in this study are openly available in the NCBI repository with accession numbers: OR652687, OR790122.

## Supplementary materials

Supplementary material 1 Secondary structures of 22 tRNA genes of *Anagyrusgalinae* Data type jpg Explanation note Blue gene names indicate that in the major strand, and red names indicate that in the minor strand. https://binary.pensoft.net/file/1085615This dataset is made available under the Open Database License (http://opendatacommons.org/licenses/odbl/1.0/). The Open Database License (ODbL) is a license agreement intended to allow users to freely share, modify, and use this Dataset while maintaining this same freedom for others, provided that the original source and author(s) are credited.Cheng-Hui Zhang, Hai-Yang Wang, Yan Wang, Zhi-Hao Chi, Yue-Shuo Liu, Guo-Hao Zu

Supplementary material 2 Secondary structures of 22 tRNA genes of *Anagyrusjenniferae* Data type jpg Explanation note Blue gene names indicate that in the major strand, and red names indicate that in the minor strand. https://binary.pensoft.net/file/1085616This dataset is made available under the Open Database License (http://opendatacommons.org/licenses/odbl/1.0/). The Open Database License (ODbL) is a license agreement intended to allow users to freely share, modify, and use this Dataset while maintaining this same freedom for others, provided that the original source and author(s) are credited.Cheng-Hui Zhang, Hai-Yang Wang, Yan Wang, Zhi-Hao Chi, Yue-Shuo Liu, Guo-Hao Zu
